# Fabrication of Au-Ag nanocage@NaYF_4_@NaYF_4_:Yb,Er Core-Shell Hybrid and its Tunable Upconversion Enhancement

**DOI:** 10.1038/srep41079

**Published:** 2017-01-20

**Authors:** Xu Chen, Donglei Zhou, Wen Xu, Jinyang Zhu, Gencai Pan, Ze Yin, He Wang, Yongsheng Zhu, Cui Shaobo, Hongwei Song

**Affiliations:** 1 State Key Laboratory on Integrated Optoelectronics, College of Electronic Science and Engineering, Jilin University, 2699 Qianjin Street, Changchun, 130012, P.R. China; 2 School of Chemical and Biomedical Engineering, Nanyang Technological University, 70 Nanyang Drive, 637457, Singapore; 3 College of Physics, Jilin University, 2699 Qianjin Street, Changchun, 130012, P.R. China; 4 Department of Physics, Nanyang Normal University, Nanyang, 473000, P.R. China; 5 Department of Physics, Nanyang Institute of Technology, Nanyang, 473000, P.R. China

## Abstract

Localized electric filed enhancement by surface plasmon resonance (SPR) of noble metal nanoparticles is an effective method to amplify the upconversion luminescence (UCL) strength of upconversion nanoparticles (UCNPs), whereas the highly effective UCL enhancement of UCNPs in colloids has not been realized until now. Here, we designed and fabricated the colloidal Au-Ag nanocage@NaYF_4_@NaYF_4_:Yb,Er core-shell hybrid with different intermediate thickness (NaYF_4_) and tunable SPR peaks from visible wavelength region to NIR region. After the optimization of the intermediate spacer thickness (~7.5 nm) of NaYF_4_ NPs and the SPR peak (~950 nm) of noble metal nanoparticles, an optimum enhancement as high as ~25 folds was obtained. Systematic investigation indicates that UCL enhancement mainly originates from the influence of the intermediate spacer and the coupling of Au-Ag nanocages with the excitation electromagnetic field of the UCNPs. Our findings may provide a new thinking on designing highly effective metal@UCNPs core-shell hybrid in colloids.

Rare-earth (RE) doped upconversion nanoparticles (UCNPs), with the larger anti-Stokes shift, higher photochemical stability, lower biotoxicity, particularly converting two or more near-infrared photons into one visible photon emission^1^, have demonstrated extensive potential applications in the fields of *in vivo* fluorescence imaging^2^,^3^,^4^, bio-sensing^5^,^6^, infrared photo-dynamical therapeutics^7^,^8^, photoelectric devices^9^ and so on. However, the lower upconversion luminescent (UCL) efficiency (less than ~3% for ~20 nm NaYF_4_:Yb^3+^,Er^3+^) as well as smaller absorption cross section for the 4*f*-4*f* transitions of UCNPs (~10^−20^ cm^−2^ at 980 nm for Yb^3+^ ions) have largely hindered their practical applications^10^. As known, localized electric filed enhancement by SPR of noble metal nanoparticles (NPs) is an effective method to amplify the luminescence strength of UCNPs, including excitation field enhancement and the increase of radiative rate (Purcell effect)^11^,^12^,^13^,^14^,^15^. In the past few years, luminescence intensity of UCNPs has been largely improved in the film devices of UCNPs/metal hybrid^16^,^17^,^18^. Our group obtained more than three-order UCL enhancement in NaYF_4_:Yb^3+^,Er^3+^ UCNPs/Au nanorods/photonic crystals hybrid^19^. He etc. reported four-order UC improvement of ZrO_2_:Yb^3+^,Er^3+^ via coupling with Au nanorods at the single particle level^20^.

Although various UCNPs@metal core-shell structures have been designed to boost UCL in colloids, however, the luminescent enhancements for those hybrid UCNPs are not obvious in colloidal solutions^21^,^22^,^23^,^24^,^25^. The enhancement factors in those hybrids usually concentrated in several times or even quenched. For instance, Zhang group observed ~4 times enhancement with optimized SiO_2_ thickness of 30 nm in Ag@SiO_2_@Y_2_O_3_:Er nanocomposites^22^. Kennedy *et al*. designed NaYF_4_:Yb,Er/Tm (inner, 20–40 nm)@Au (outer, 4–8 nm) core-shell structure, and demonstrated ~8 times enhancment^23^. Duan and co-workers also reported an enhancement factor of 2.5 by the attachment of the gold NPs onto NaYF_4_:Yb^3+^,Tm^3+^ NPs, while the formation of the continuous gold shell onto NaYF_4_:Yb^3+^,Tm^3+^ NPs could quench the emission^25^.

Compared with UCL enhancement in the film, the extremely low UC enhancement in the colloids could be attributed to the following several reasons. First, most of previous works in colloids designed the UCNPs (inner)@metal (outer) core-shell hybrid, which would greatly reduce the energy of excitation light illuminated on UCNPs owing to the scattering and absorption of metal shell to excitation light^23^,^26^. Second, according to previous literatures^16^,^26^, the enhancement of excitation electric field rather than the Purcell effect is the dominating factor for inducing more intense improvement of UCL, which means the location of surface plasmon resonance (SPR) of metal nanostructures should be tuned to excitation wavelength. However, the SPR peaks usually were not expanded to the near infrared region (NIR) in UCNPs@metal hybrid colloids (~980 nm for UCNPs). In addition, luminescent enhancement is proportional to the ratio of scattering coefficient to absorption coefficient of metal NPs^27^. Theoretical and experiment results both predicted that the improved particle size, the anisotropy and Au-Ag alloy nanostructure could improve the ratio of scattering to absorption coefficient^28^,^29^.

Taken into account of the above factors together, we designed Au-Ag nanocage@NaYF_4_@NaYF_4_:Yb,Er core-shell composite in colloids, which demonstrates tunable SPR from visible to NIR. It should be highlighted that the intermediate spacer NaYF_4_ with suitable thickness could prevent the possible energy transfer from NaYF_4_:Yb,Er to the metal nanostructure. After the optimization of the intermediate spacer thickness (~7.5 nm) of undoped NaYF_4_ and the SPR peak (~950 nm) of noble metal NPs, an optimum enhancement as high as ~25 folds was obtained.

## Results and Discussion

### Morphology and Structure of Au-Ag nanocage@NaYF_4_ @NaYF_4_:Yb,Er

**Figure 1:**
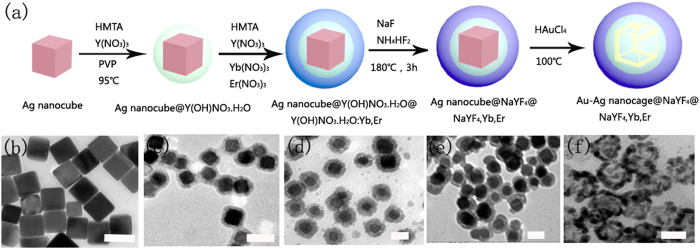
(**a**) Schematic illustrations of Au-Ag nanocage@NaYF_4_@NaYF_4_:Yb,Er formation process. (**b**–**f**) TEM images of Ag nanocube, Ag nanocube@Y(OH)NO_3_**·**H_2_O, Ag nanocube@Y(OH)NO_3_.H_2_O @Y(OH)NO_3_**·**H_2_O: Yb,Er, Ag nanocube@NaYF_4_@NaYF_4_:Yb,Er, and Au-Ag nanocage@NaYF_4_@ NaYF_4_:Yb,Er, respectively. All of scale bar: 50 nm.

Figure 1(a) presents the design and fabrication strategy of Au-Ag nanocage@NaYF_4_@ NaYF_4_:Yb,Er hybrid nanostructures. The detailed synthesis and experimental procedures are described in the experimental section. First, the uniform Ag nanocubes with an edge length of ~30 nm were prepared according to the previous work with a minor modification (Fig. 1(b))^30^,^31^. Then, the uniform Ag nanocubes were coated with a thin layer of Y(OH)NO_3_**·**H_2_O (~4 nm) by a hydrothermal route (3 h) by gradually adding polyvinylpyrrolidone (PVP), hexamethylenetetramine (HMTA) and Y(NO_3_)_3_**·**6H_2_O, where the surfactant PVP assisted to deposit the RE precursors on Ag nanocubes, and HMTA could hydrolyze in water to give NH_3_, promoting the forming of Y(OH)NO_3_**·**H_2_O (Fig. 1(c))^32^. The Energy-dispersive X-ray (EDX) mapping and analysis of Ag nanocube@Y(OH)NO_3_**·**H_2_O were detected to analysis the elements, as shown in Fig. S1(a–e). Figure S1(b–d) represent the mapping of silver, oxygen, and yttrium elements, respectively. It can be seen that oxygen, and yttrium elements distribute homogeneously on the Ag nanocubes. Otherwise, noted that the thickness of Y(OH)NO_3_**·**H_2_O layer on the Ag nanocubes can be controlled by changing the reaction time. The Ag nanocube@Y(OH)NO_3_**·**H_2_O hybrids were further coated a luminescent layer of Y(OH)NO_3_**·**H_2_O:Yb,Er by the same method with Fig. 1(d). It can be seen that after further coating Y(OH)NO_3_**·**H_2_O:Yb,Er, the shell thickness increases to ~8 nm, indicating the formation of Ag nanocube@Y(OH)NO_3_**·**H_2_O (~4 nm) @Y(OH)NO_3_**·**H_2_O,Yb,Er (~4 nm). Ag-nanocube@NaYF_4_@NaYF_4_:Yb,Er were synthesized by the fluorination through dissolving the NaF and NH_4_HF_2_ in above Ag nanocube@Y(OH)NO_3_**·**H_2_O@Y(OH)NO_3_**·**H_2_O: Yb,Er solution under the hydrothermal condition at 180 °C (Fig. 1(e))^33^. At last, Au-Ag nanocage@NaYF_4_@NaYF_4_:Yb,Er structures were prepared based on the galvanic replacement reaction between the silver nanocubes and HAuCl_4_ solution^34^. Differing from the solid Ag nanocube@NaYF_4_@NaYF_4_:Yb,Er, Fig. 1(f) presents hollow structure, heralding the formation of Au-Ag nanocages@NaYF_4_@NaYF_4_:Yb,Er. EDX analysis and X-ray diffraction (XRD) pattern of Au-Ag nanocage@NaYF_4_@NaYF_4_:Yb,Er in Fig. S2 indicate that the core-shell structure consists of cubic phase Au/Ag and cubic phase NaYF_4_.

**Figure 2:**
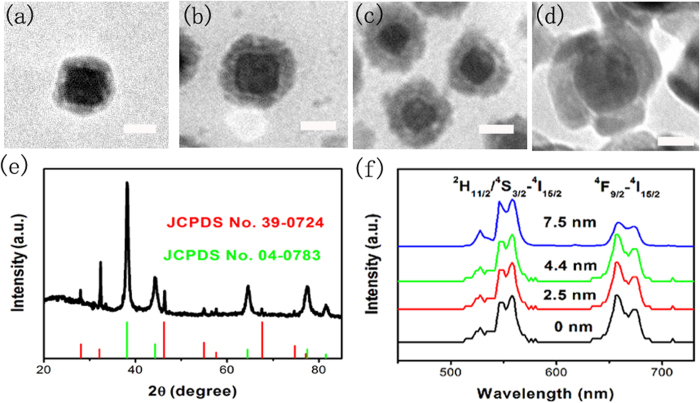
(**a–d**) TEM images of Ag nanocube@NaYF_4_@NaYF_4_:Yb,Er with unaltered thickness of NaYF_4_:Yb,Er luminescence layer (~4 nm) by tailoring different thickness of intermediate spacer NaYF_4_: (**a**) ~0 nm, (**b**) ~2.5 nm, (**c**) ~4.4 nm, (**d**) ~7.5 nm. (**e**) XRD pattern of Ag nanocube@NaYF_4_@NaYF_4_:Yb,Er and standard cards of cubic NaYF_4_ (JCPDS No. 39-0724) and Ag (JCPDS No. 04-0783). (**f**) UCL spectra of Ag nanocube@NaYF_4_@NaYF_4_:Yb,Er with different thickness (0–7.5 nm) of intermediate NaYF_4_ under 980 nm light excitation (~0.33 W). All of scale bar: 20 nm.

**Figure 3:**
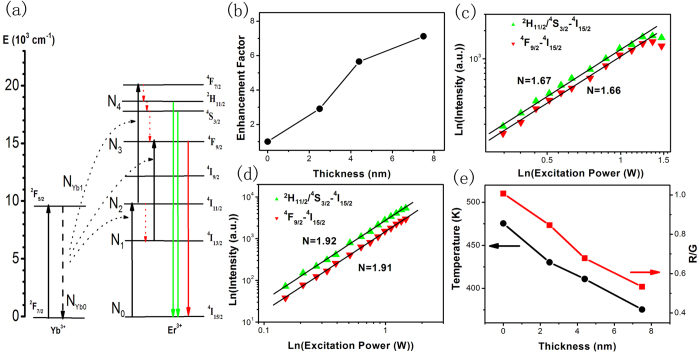
(**a**) Schematic of UC populating mechanism for NaYF_4_:Yb,Er under 980 nm excitation. (**b**) EF of Ag nanocube@NaYF_4_@NaYF_4_:Yb,Er versus different intermediate NaYF_4_ thickness, comparing with Ag nanocube@NaYF_4_:Yb,Er, (The excitation power of ~0.33 W). (**c,d**) Power dependent UCL intensity of different transitions in Ag nanocube@NaYF_4_:Yb,Er and Ag nanocube@7.5 nm NaYF_4_@NaYF_4_:Yb,Er, respectively. (**e**) Temperature calculated based on branch ratio (*R*_*HS*_) of ^2^H_11/2_-^4^I_15/2_ to ^4^S_3/2_-^4^I_15/2_ transitions, and the ratio between red emission and green emission (R/G) in different Ag-nanocube@NaYF_4_@NaYF_4_:Yb,Er samples versus different intermediate NaYF_4_ thickness.

Generally, direct contact of metal NPs with UCNPs would induce luminescent quenching due to the energy transfer from the UCNPs to metal NPs^22^. To prevent this negative energy transfer, we designed an intermediate spacer with homogeneous NaYF_4_ which has the complete lattice matching with NaYF_4_:Yb,Er luminescent layer, and is helpful of precise controlling the thickness of intermediate spacer. As shown in Fig. 2(a–d), the thicknesses of intermediate spacer NaYF_4_ in different reaction time are determined to 0, 2.5 nm, 4.4 nm, and 7.5 nm in Ag nanocube@NaYF_4_@NaYF_4_:Yb,Er hybrid nanostructures, respectively. These thicknesses of intermediate spacer NaYF_4_ remain unchanged compared to the thicknesses of Y(OH)NO_3_**·**H_2_O layer of Ag nanocube@Y(OH)NO_3_**·**H_2_O after the hydrothermal reaction, as shown in Fig. S3(a–c), (a) ~2.6 nm, (b) ~4.3 nm, (c) ~7.8 nm. Noted that the thickness of NaYF_4_:Yb,Er luminescent layer remains unaltered (~4 nm) in different samples. The XRD pattern further confirms the formation of Ag nanocube @NaYF_4_@NaYF_4_:Yb,Er (Fig. 2(e)), where the diffraction peaks agree well with the cubic phase NaYF_4_ (red line), and the cubic phase Ag (green line), respectively. The normalized UCL spectra of Ag-nanocube@NaYF_4_@NaYF_4_:Yb,Er with different thickness of intermediate NaYF_4_ under 980 nm light excitation are shown in Fig. 2(f). The two dominating Er^3+^ emission peaks at 550 nm and 660 nm are assigned to the transitions of ^2^H_11/2_/^4^S_3/2_-^4^I_15/2_ and ^4^F_9/2_-^4^I_15/2_, respectively. Interestingly, the red emission (^4^F_9/2_-^4^I_15/2_) decreases with increasing the intermediate NaYF_4_ thickness. The populating of red level is related to the nonradiative transition of ^4^S_3/2_-^4^F_9/2_ or ^4^I_11/2_-^4^I_13/2_, as shown in Fig. 3(a). The direct attaching of Ag-nanotube with NaYF_4_:Yb^3+^,Er^3+^ would alter the nonradiative relaxation rate, leading to the relative intensity increase of red to green. The involving of intermediate spacer would prevent the nonradiative relaxation to some extent. The intermediate spacer thickness-dependent spectra reveal that the emission intensity is sensitive to the distance between the Ag nanocubes and NaYF_4_:Yb,Er shell.

### UCL enhancement and dynamics of Ag nanocube@NaYF_4_@ NaYF_4_:Yb,Er

A schematic illustration on the UC populating and emission processes of NaYF_4_:Yb,Er is shown in Fig. 3(a), where the green and red emissions both come from two-photon processes^1^. Figure 3(b) shows the enhancement factor (EF) as a function of the intermediate NaYF_4_ thickness ranging of 0–7.5 nm, compared with the Ag nanocube@NaYF_4_:Yb,Er nanostructure. It can be seen that the EF increases with increasing of the intermediate NaYF_4_ thickness, and reaches a maximum value of ~7.2 fold at an optimal intermediate spacer thickness of ~7.5 nm. In general, the energy transfer efficiency from emitters to Ag nanocubes should be inversely proportional to their distance, which causing quenching of emitters. Therefore, with the increase thickness of intermediate spacer, the energy transfer from Er^3+^ ions to Ag nanocubes decreases, leading to the improvement of UCL^14^,^22^.

Figure 3(c) and (d) record the double-logarithmic plots of the power dependent UCL intensity of ^2^H_11/2_/^4^S_3/2_-^4^I_15/2_ and ^4^F_9/2_-^4^I_15/2_ transitions for Ag nanocube@ NaYF_4_:Yb,Er and Ag nanocube@7.5 nm NaYF_4_@NaYF_4_:Yb,Er, respectively. The slops of Ag nanocube@NaYF_4_:Yb,Er without intermediate spacer are smaller than those of the intermediate spacer 7.5 nm NaYF_4_, which are close to the required photon numbers (n = 2) to populate the corresponding levels. This can be mainly attributed to the local thermal effect, originating from the medium absorption, photo-thermal conversion to excitation light and the photo-thermal effect of Ag nanocubes, leading to the increase of non-radiative process and luminescent quenching^35^,^36^. To further demonstrate the local thermal effect, we calculate the temperature according to the intensity branch ratio (*R*_*HS*_) of ^2^H_11/2_-^4^I_15/2_ to ^4^S_3/2_-^4^I_15/2_ transitions (see S1 and Fig. S4). It is obvious that the temperature decreases with the increasing of the intermediate spacer thickness (Fig. 3(e)). This indicates that the photo-thermal effect have great impact on the variation of EF with the intermediate spacer thickness. Meanwhile, the temperature increase in Ag nanocube@NaYF_4_@ NaYF_4_:Yb,Er with thinner spacer leads to the increase of the non-radiative rate (such as ^4^S_3/2_-^4^F_9/2_, ^4^I_11/2_-^4^I_13/2_), generating the higher ratio between red emission and green emission (R/G) (Figs 2(f) and 3(e)).

**Figure 4:**
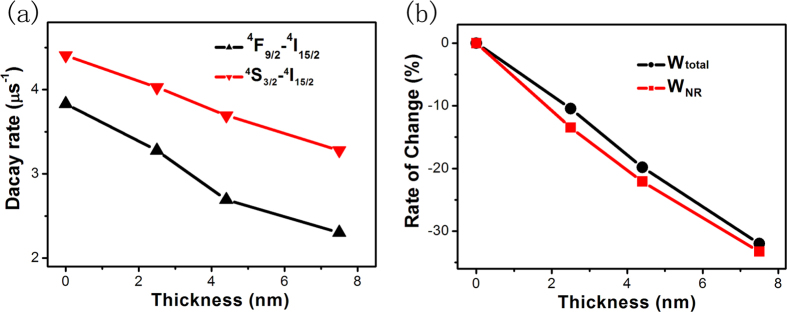
(**a**) Decay rate of ^4^S_3/2_-^4^I_15/2_ and ^4^F_9/2_-^4^I_15/2_ as a function of the intermediate NaYF_4_ thickness. (**b**) Variation of calculated nonradiative relaxation rate (*W*_*NR*_, ^4^F_7/2_-^2^H_11/2_/^4^S_3/2_) and the total decay rate (*W*_*total*_, ^4^S_3/2_-^4^I_15/2_) as a function of the intermediate spacer thickness.

To further determine the mechanism of UCL enhancement, the decay dynamics of Ag nanocube@NaYF_4_@NaYF_4_:Yb,Er hybrid nanostructures were investigated under the excitation of 980-nm pulsed laser, as shown in Fig. 4(a) and Fig. S5. Through fitting the decay curves (Fig. S5) with the single exponential function, Fig. 4(a) shows the calculated UCL decay rates of ^4^S_3/2_-^4^I_15/2_ and ^4^F_9/2_-^4^I_15/2_ transitions as a function of the intermediate NaYF_4_ thickness. It indicates that the UCL decay rate of ^4^S_3/2_-^4^I_15/2_ and ^4^F_9/2_-^4^I_15/2_ transitions decreases with the increasing of the intermediate NaYF_4_ thickness, and the variation of total decay rate (*W*_*total*_) is only 30% (Fig. 4(b)). To figure out the essence for the variation of *W*_*total*_, the temperature-dependent nonradiative relaxation rate (*W*_*NR*_) of ^4^F_7/2_-^2^H_11/2_/^4^S_3/2_ was calculated by the multi-photon theory (Figs 4(b), and S2). The result demonstrates that as the temperature varies ranging of several ten centigrade (see Fig. 3(e)), the variation of W_NR_ is also about 30% (Fig. 4(b)). This definitely reveals that the variation of the total transition rate (*W*) probably originates from the change of *W*_*NR*_. That is the reason that the photo-thermal effect of Ag nanocubes decreases with the increasing of intermediate NaYF_4_ thickness. This implies that the variation of *W*_*total*_ in Ag-nanocube@NaYF_4_@NaYF_4_:Yb,Er with different thickness spacer probably originates from the change of *W*_*NR*_ instead of the radiative rate. This fact indicates that the upconversion enhancement can be mainly attributed to the coupling of SPR with the excitation light, instead of Purcell effect, which alters the radiative transition rate. Overall, the intermediate layer (NaYF_4_) could prevent the energy transfer from Er^3+^ ions to Ag nanocube and decrease the thermal diffusion from Ag nanocube to luminescent layer (NaYF_4_:Yb,Er), leading to the higher UCL enhancement with thicker spacer.

### UCL enhancement and mechanism of Au-Ag nanocage@NaYF_4_ @NaYF_4_:Yb,Er

**Figure 5:**
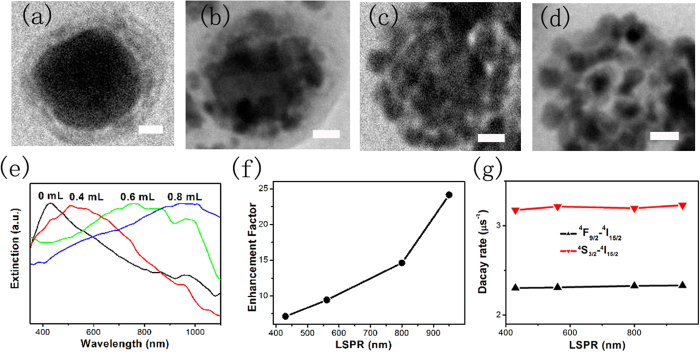
(**a–d**) TEM images of Ag nanocube@7.5 nm NaYF_4_@NaYF_4_:Yb,Er after the galvanic replacement reaction with different volumes (0, 0.4, 0.6, 0.8 mL) of 0.3 mM HAuCl_4_ solution. (**e**) Extinction spectra corresponding to the TEM images of (**a–d**). (**f**) EF of Au-Ag nanocage@7.5 nm NaYF_4_@NaYF_4_:Yb,Er versus the LSPR peaks, related to the Ag nanocube@7.5 nm NaYF_4_@NaYF_4_:Yb,Er. (g) Decay rates of ^4^S_3/2_-^4^I_15/2_ and ^4^F_9/2_-^4^I_15/2_ as a function of the LSPR locations. All of scale bar: 10 nm.

The localized surface plasmon resonance (LSPR) peak has profound influence on UCL of the UCNPs. Figure 5(a–d) shows the representative TEM images with different volumes (0, 0.4, 0.6, 0.8 mL) of 0.3 mM HAuCl_4_ solution. It can be seen that Ag nanocubes gradually transform to Au-Ag nanocages with uniform walls and hollow interiors. When the volume of the HAuCl_4_ solution increases to 0.8 mL, Au-Ag nanocages collapse into gold fragments, indicating the initiation of dealloying^34^. The SPR peak of the Au-Ag nanocages changes from 430 nm to 950 nm, matching well with the excitation wavelength (λ = 980 nm), corresponding to the alloying and dealloying process (see Fig. 5(e)).

EFs of Au-Ag nanocage@7.5 nm NaYF_4_@NaYF_4_:Yb,Er nanostructures at different LSPR peaks were recorded, as shown in Fig. 5(f). It can be seen that as the LSPR peaks shifts to red, the EF of UCL increases gradually. An optimum EF of ~25 fold is obtained when the LSPR peak is tuned to 950 nm, closing to the excitation wavelength (980 nm). This finding suggests that the UCL enhancement could be attributed to the coupling of LSPR with the excitation field, leading to strength enhancement of local excitation field. In addition, the decay dynamics of Au-Ag nanocage@NaYF_4_ @NaYF_4_:Yb,Er hybrids with different LSPR peaks were studied under the excitation of 980-nm pulsed laser. Fig. 5(g) shows the UCL decay rate of ^4^S_3/2_-^4^I_15/2_ and ^4^F_9/2_-^4^I_15/2_ transitions as a function of the LSPR peaks, respectively. The decay rates remain unaltered, which also indicates that the upconversion enhancement in Au-Ag nanocage@NaYF_4_@NaYF_4_:Yb,Er with different LSPR peaks originates from the coupling of SPR with the excitation light, similarly in Ag nanocube@NaYF_4_@ NaYF_4_:Yb,Er with different thickness spacer.

## Conclusions

In this work, we fabricated the Ag nanocube@NaYF_4_@NaYF_4_:Yb,Er hybrid with different intermediate spacer thickness (0–7.5 nm), and Au-Ag nanocage@NaYF_4_@NaYF_4_:Yb,Er with tunable SPR peaks from visible region to NIR region (430–950 nm). It is found that UCL enhancement in Ag nanocube@NaYF_4_@NaYF_4_:Yb,Er increases with increasing of the intermediate NaYF_4_ thickness, which is attributed to the inhibition of energy transfer from Er^3+^ to Ag nanocubes and the inhibition of thermal expansion from Ag nanocubes to Er^3+^. After optimization, the UCL enhancement of ~25 times are obtained (intermediate spacer with the thickness of ~7.5 nm and LSPR peak at ~950 nm). The detail investigation indicates that the UCL enhancement mainly originates from the coupling of Ag nanocubes or Au-Ag nanocages with the excitation electromagnetic field of the UCNPs. Overall, our work may provide a new thinking on designing a highly effective metal@UCNPs core-shell structure in colloids, which is expected to applied to bioapplications and photoelectric devices.

## Experimental Section

### Materials

Silver nitrate (AgNO_3_, Alfa Aesar), chloroauric acid (HAuCl_4_, Aladdin), deionized (DI) water, polyvinylpyrrolidone (PVP), ammonium acid fluoride (NH_4_HF_2_, Fuchen Chemical Reagents), sodium sulfide (Na_2_S**·**9H_2_O, Beijing Chemical Works), sodium fluoride (NaF, Fuchen Chemical Reagents), hexamethylenetetramine (HMTA. Beijing Chemical Works), sodium chloride (NaCl, Fuchen Chemical Reagents), ethylene glycol (EG, Beijing Chemical Works), ethanol (Beijing Chemical Works), yttrium (III) nitrate hexahydrate (Y(NO_3_)_3_.6H_2_O, Ruike Centre), ytterbium nitrate hexahydrate (Yb(NO_3_)_3_.6H_2_O, Ruike Centre), and erbium nitrate hexahydrate (Er(NO_3_)_3_.6H_2_O, Ruike Centre) were used in our work without further purification.

### Synthesis of Ag nanocubes

Silver nanocubes with a edge length of ~30 nm were prepared according to the previous report with a minor modification^30^,^31^. An amount of 230 mL of EG was added into a three-neck round bottomed flask and heated to 150 °C under vigorous magnetic stirring in an oil bath, and then kept for another 60 min. An amount of 2.8 mL of Na_2_S**·**9H_2_O (3 mM in EG) was injected into the EG solution, followed by adding 60 mL of PVP (Mw = 29000, 20 mg/mL in EG) with a separated funnel under capped conditions. After the temperature was increased to 180 °C, 20 mL of AgNO_3_ (48 mg/mL in EG) was added dropwise into the above solution. After the addition of AgNO_3_, the color of reaction solution changed from yellow to green ochre and brown within 30 min. Then, the solution was cooled to room temperature in an ice bath. The as-synthesized samples were washed with water for three times using centrifugation, and dissolved in 10 mL of an aqueous solution.

### Synthesis of Ag nanocube@Y(OH)NO_3_ **·**H_2_O

First, 1.0 mL of above Ag nanocube/water solution was dissolved in the 100 mL of an aqueous solution. Then PVP (Mw = 29000, 110 mM) solution was directly added to the above solution. The mixture solution of PVP and seed Ag nanocubes was vortexed for 5 s, followed by the addition of HMTA (0.5 mM) and, Y(NO_3_)_3_**·**6H_2_O (0.5 mM). After vortexing for 10 s, the reaction mixture was incubated at 95 °C. Finally, Ag nanocube@Y(OH)NO_3_**·**H_2_O was formed, and the thickness of Y(OH)NO_3_.H_2_O was controlled via adjusting the reaction time (2–5 h). As-synthesized Ag nanocube@Y(OH)NO_3_.H_2_O was washed for three cycles using centrifugation and redissolved in 100 mL water.

### Synthesis of Ag nanocube@Y(OH)NO_3_ **·**H_2_O@Y(OH)NO_3_ **·**H_2_O: Yb,Er

Half of the above solution (Ag nanocube@Y(OH)NO_3_**·**H_2_O) was added the PVP (110 mM) and HMTA (0.5 mM). Then, 0.5 mM of Y(NO_3_)_3_.6H_2_O(78%), Yb(NO_3_)_3_**·**6H_2_O(20%), and Er(NO_3_)_3_**·**6H_2_O(2%) were added. After vortexing for 10 s, the reaction mixture was incubated at 95 °C for 3 h. The product of Ag nanocube@ Y(OH)NO_3_**·**H_2_O@Y(OH)NO_3_**·**H_2_O:Yb,Er were washed for three cycles using centrifugation.

### Synthesis of Ag nanocube@NaYF_4_@NaYF_4_:Yb,Er

The as-obtained Ag nanocube@Y(OH)NO_3_**·**H_2_O@Y(OH)NO_3_**·**H_2_O:Yb,Er was dispersed into 10 mL of ethanol. Then 0.42 mg of NaF and 2.28 mg NH_4_HF_2_ were dripped into the suspension under stirring. After additional agitation for 30 min, the as-obtained mixture was transferred into a 20 mL autoclave and heated at 180 °C for 3 h. After cooling the above solution to room temperature, the as-prepared Ag nanocube@NaYF_4_@NaYF_4_:Yb,Er were further washed for three cycles using centrifugation.

### Synthesis of Au-Ag nanocage@NaYF_4_@NaYF_4_:Yb,Er

The Au-Ag nanocage@NaYF_4_@NaYF_4_:Yb,Er nanostructures were prepared based on the galvanic replacement reaction between the silver nanocubes and HAuCl_4_^34^. First, Ag nanocube@NaYF_4_ @NaYF_4_:Yb,Er hybrid nanostructures was diluted with 10 mL of deionized water and heated to 100 °C. Then, the different volumes (0.2−0.8 mL) of HAuCl_4_ (0.3 mM) solution were added dropwise and refluxed for 10 min until its color became stable. After cooling to room temperature, NaCl solid was added into the mixture until the solution was saturated to dissolve the AgCl product. Finally, the Au-Ag nanocage@NaYF_4_@NaYF_4_:Yb,Er nanostructures were separated by centrifugation at 12000 rpm for 15 min and washed with water several times.

### Characterization

The HR-TEM images were recorded on a JEOL-2100F high resolution transmission electron microscope under a working voltage of 200 kV. Energy-dispersive X-ray (EDX) analysis of the samples was also carried out during HR-TEM measurements to obtain the elemental analysis of samples. The phase structure and purity of the as-prepared samples were characterized by X-ray power diffraction (XRD) with a Rigaku D/max 2550 X-ray diffractometer, using a monochromatized Cu target radiation resource (λ = 1.54 Å). Ultraviolet-visible (UV-Vis) extinction spectra were measured with a Shimadzu UV-3101PC UV-vis scanning spectrophotometer ranging of 300–1100 nm.

### Optical measurement

The UCL spectra were measured using an Andor Shamrock SR-750 spectrometer. A photomultiplier combined with a monochromator was used for signal collection from 300 nm to 750 nm. A continuous 980 nm diode laser was used to pump the samples to investigate the steady-state spectra. In the measurements of luminescent dynamics, the samples were pumped by a laser-system consisting of a Nd:YAG pumping laser (1064 nm), the third-order Harmonic-Generator (355 nm) and a tunable optical parameter oscillator (OPO, Continuum Precision II 8000). It was with the pulse duration of 10 ns, repetition frequency of 10 Hz and line width of 4–7 cm^−1^.

## Additional Information

**How to cite this article**: Chen, X. *et al*. Fabrication of Au-Ag nanocage@NaYF_4_@NaYF_4_:Yb,Er Core-Shell Hybrid and its Tunable Upconversion Enhancement. *Sci. Rep.* **7**, 41079; doi: 10.1038/srep41079 (2017).

**Publisher's note:** Springer Nature remains neutral with regard to jurisdictional claims in published maps and institutional affiliations.

## Supplementary Material

Supplementary Information
