# Supplementary material for: Fabrication of Au-Ag nanocage@NaYF4@NaYF4:Yb,Er Core-Shell Hybrid and its Tunable Upconversion Enhancement
Source: Sci Rep. 2017 Jan 20;7:41079. doi: 10.1038/srep41079 (PMC5247729; doi:10.1038/srep41079)
Supplement: Supplementary Information [file srep41079-s1.pdf]

Supplementary Information

Fabrication of Au-Ag nanocage@$NaYF_4$@$NaYF_4$:Yb,Er Core-Shell Hybrid and its Tunable Upconversion Enhancement

Xu Chen[1], Donglei Zhou[1], Wen Xu[1,3]*, Jinyang Zhu[2], Gencai Pan[1], Ze Yin[1], He Wang[2], Yongsheng Zhu[4], Shaobo, Cui[5], Hongwei Song[1]*

[1]State Key Laboratory on Integrated Optoelectronics, College of Electronic Science and Engineering, Jilin University, 2699 Qianjin Street, Changchun, 130012, P. R. China. [2]College of Physics, Jilin University, 2699 Qianjin Street, Changchun, 130012, P. R. China. [3]School of Chemical and Biomedical Engineering, Nanyang Technological University, 70 Nanyang Drive, Singapore 637457. [4]Department of Physics, Nanyang Normal University, Nanyang, 473000, P. R. China. [5]Department of Physics, Nanyang Institute of Technology, Nanyang, 473000, P. R. China.

*E-mail: wen_xu09@163.com, songhw@jlu.edu.cn

S1 Calculation of the sample temperature

Based on the Boltzmann thermal distribution,[1] the intensity ratio (R_{HS}) of $^2H_{11/2}$-$^4I_{15/2}$ to $^4S_{3/2}$-$^4I_{15/2}$ can be deduced that

$$R_{HS} = R(0)\exp(-\Delta E / KT) \quad (1)$$

where $R(0)$ is a constant, ΔE is the energy separation between the $^2H_{11/2}$ and $^4S_{3/2}$ levels (~790 cm^{-1}), K is Boltzmann's constant, and T is the absolute temperature. As the excitation power is low, the sample temperature at the pumped spot should be close to the room temperature (~293 K). Therefore, *R(0)* could be obtained from the experimental data of the corresponding R_{HS} value. Based on the experimental relationship of R_{HS} and pumped power density, the temperature T at the irradiated spot as a function of pump power density can be deduced, as shown in Fig.S2 .

S2 Calculation of the nonradiative relaxation rate

Based on the multi-phonon relaxation theory, the nonradiative relaxation rate with temperature can be written as

$$W_{NR}(T) = W_{NR}(0)[1 - \exp(-\hbar\omega / kT)]^{-\Delta E/\hbar\omega} \qquad (2)$$

where $W_{NR}(0)$ presents the nonradiative relaxation rate at absolute zero, $\hbar\omega$ is the average phonon energy, k is Boltzmann's constant, T is the absolute temperature in K, and ΔE is the energy difference between upper and lower energy levels from which nonradiative relaxation occurs. In our case, the energy difference (ΔE) between $^4F_{7/2}$ and its nearest down level $^2H_{11/2}$ is ~1170 cm^{-1} for the two-photon populating $^4F_{7/2}$-$^2H_{11/2}$/ $^4S_{3/2}$ transition. and phonon energy ($\hbar\omega$) of $NaYF_4$ host is in the vicinity of ~350 cm^{-1}. The variation of W_{NR} with the temperature could be carried out, as shown in Fig.3e.

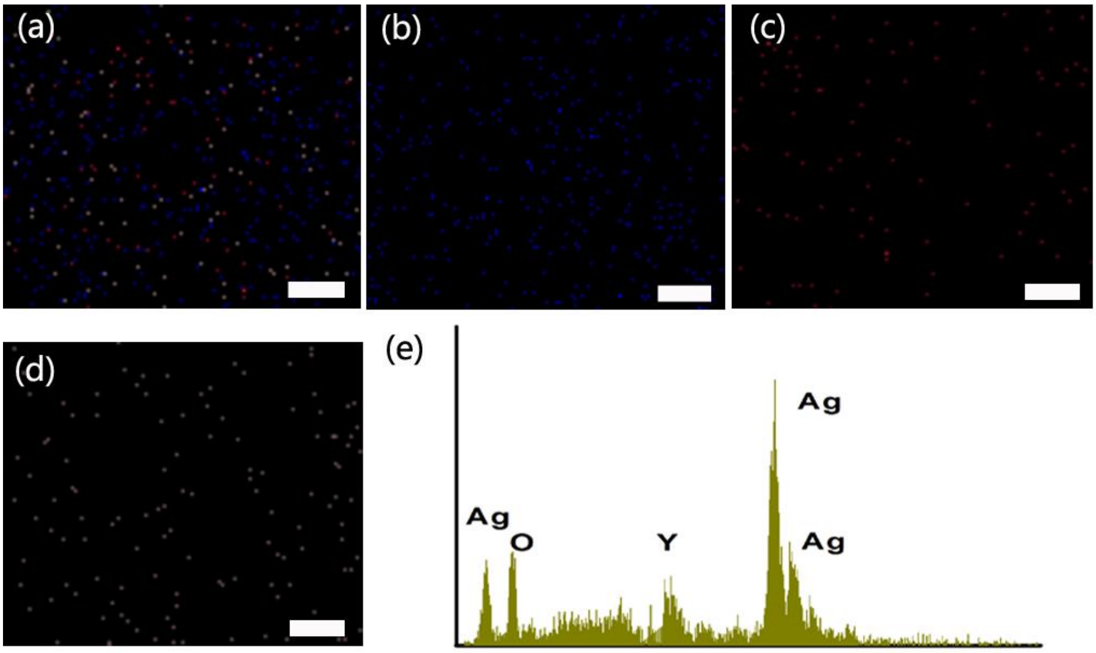

Figure S1 (a-d) the energy-dispersive X-ray (EDX) mapping of Ag nanocube@Y(OH)NO_3•H_2O: silver(b), oxygen(c), and yttrium(d) elements; (e) EDX analysis of Ag nanocube@Y(OH)NO_3•H_2O. All of scale bar: 20 nm.

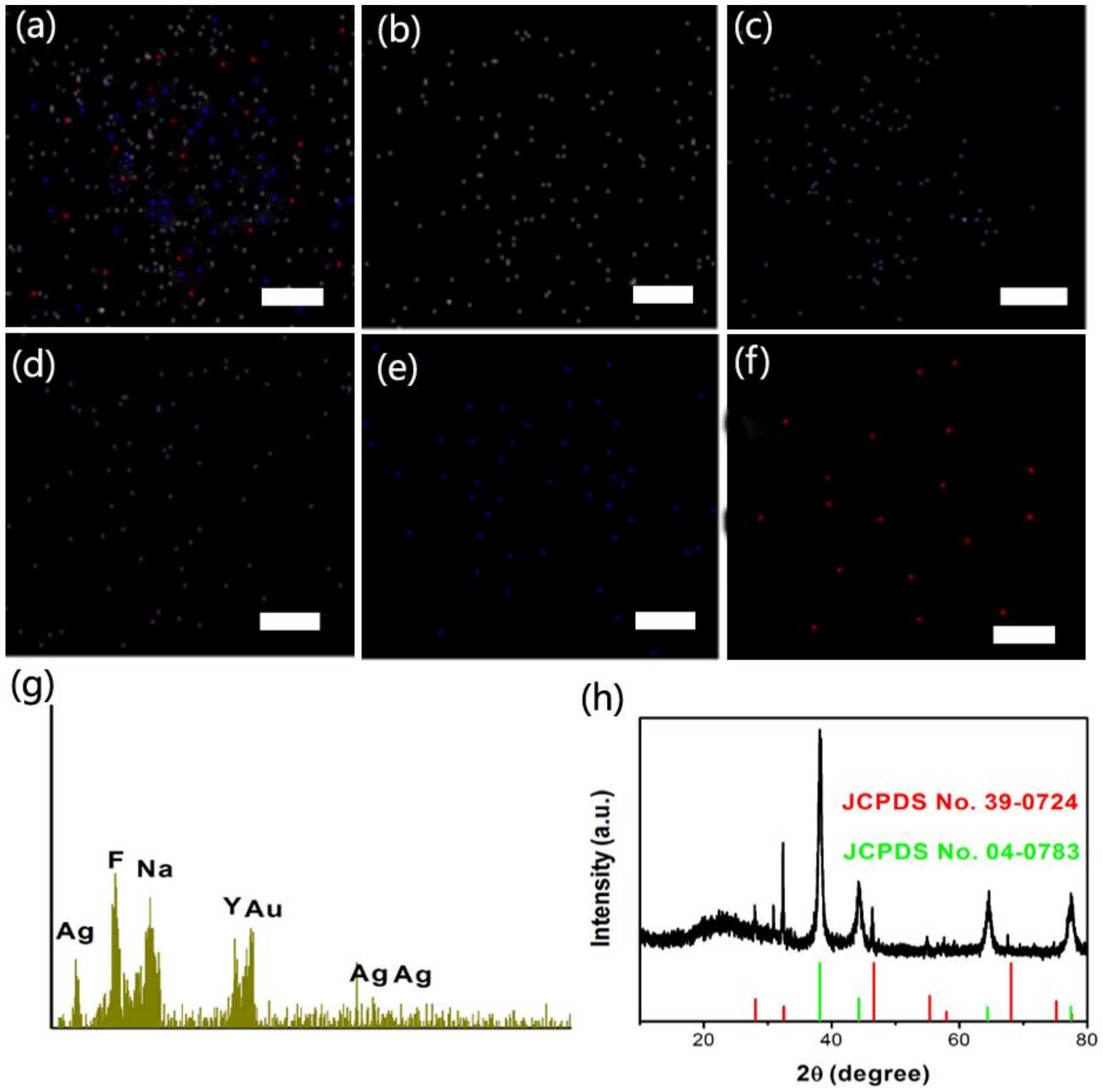

Figure S2 (a-f) EDX mapping of Au-Ag nanocage@$NaYF_4$@$NaYF_4$:Yb,Er: fluorine(b), yttrium(c), sodium(d), gold(e) and silver(f) elements; (g) EDX mapping analysis of Au-Ag nanocage@$NaYF_4$@$NaYF_4$:Yb,Er, (h) XRD pattern of Au-Ag nanocage@$NaYF_4$@$NaYF_4$:Yb,Er and the standard of cubic NaYF4 (JCPDS No. 39-0724) and cubic Ag (JCPDS No. 04-0784). All of scale bar: 20 nm.

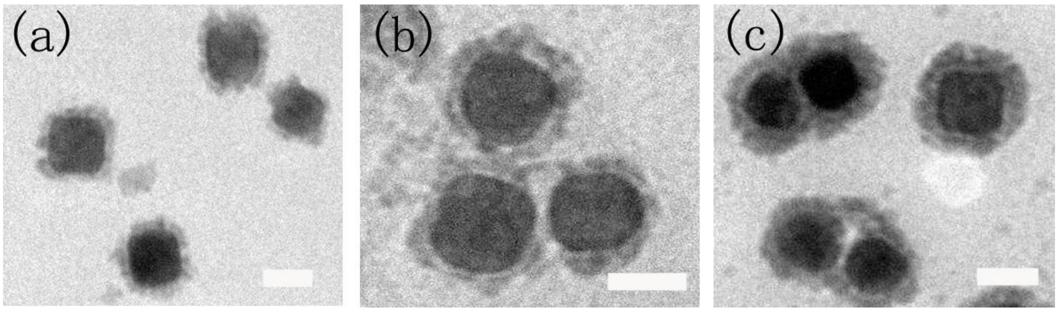

Figure S3 TEM images of Ag nanocube@Y(OH)NO_3•H_2O with different thickness of Y(OH)NO_3•H_2O layer (a) ~2.6 nm, (b) ~4.3 nm, (c) ~7.8 nm. All of scale bar: 20 nm.

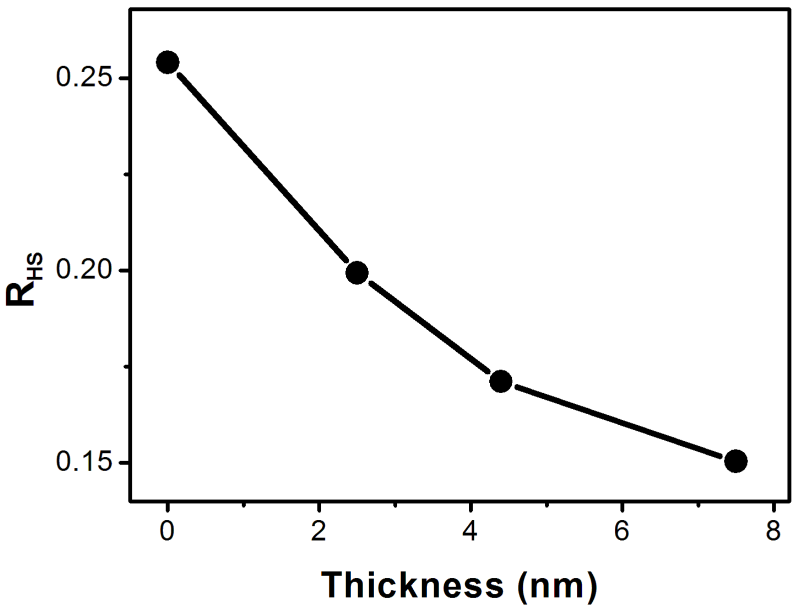

Figure S4 Thickness dependence of branch ratio (R_{HS}) of $^2H_{11/2}$-$^4I_{15/2}$ to $^4S_{3/2}$-$^4I_{15/2}$ transitions in Ag nanocube@$NaYF_4$@$NaYF_4$:Yb,Er hybrid nanostructures.

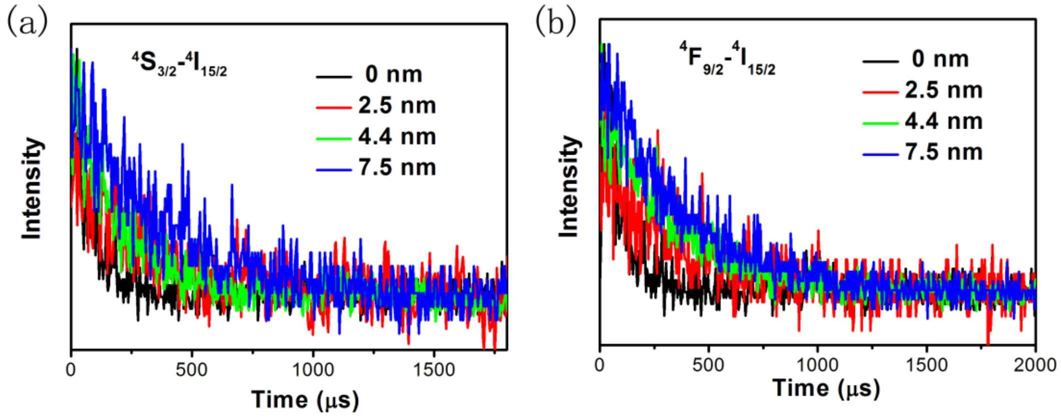

Figure S5 UCL decay curves of $^4S_{3/2}$-$^4I_{15/2}$(a) and $^4F_{9/2}$-$^4I_{15/2}$(b) of Ag nanocube@$NaYF_4$@$NaYF_4$:Yb,Er with different intermediate $NaYF_4$ thickness under 980 nm excitation.

References

1. Dai Q. L. et al. Structure and upconversion luminescence of hydrothermal $PbWO_4$: Er^{3+}, Yb^{3+} powders. *J. Phys. Chem. C* **112**, 19694–19698 (2008).
